# Supplementary material for: COVID-19 and influenza infections mediate distinct pulmonary cellular and transcriptomic changes
Source: Commun Biol. 2023 Dec 13;6:1265. doi: 10.1038/s42003-023-05626-z (PMC10719262; doi:10.1038/s42003-023-05626-z)
Supplement: Supplementary file 2 — Supplementary information [file 42003_2023_5626_MOESM2_ESM.pdf]

Supplemental Figure 1

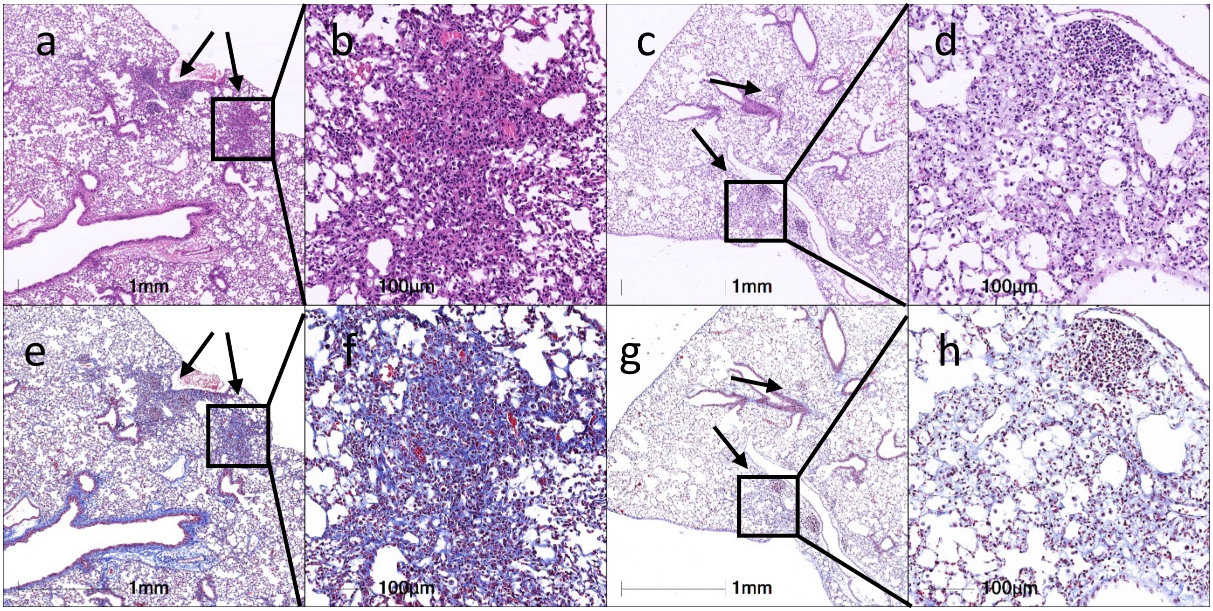

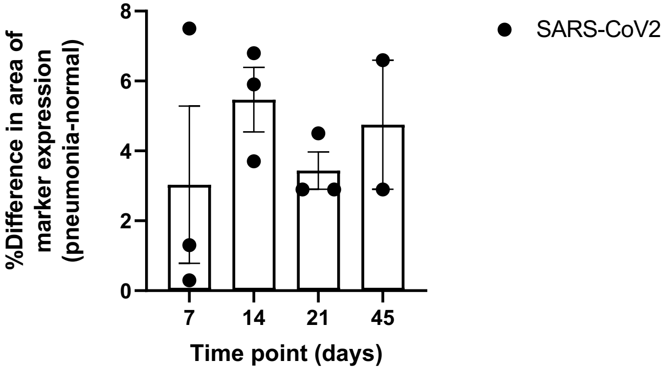

Supplemental Figure 1. Persistent pulmonary pathology in SARS-CoV-2 infected mice out to 45 days post infection (n=2). a-d) At 45 DPI there are persistent, patchy regions of pulmonary consolidation with inflammation (arrows) in SARS-CoV-2 infected mice (a&b Ms 307; c&d Ms 314). e-h) Trichrome staining reveals pulmonary fibrosis without epithelial proliferation in regions of pulmonary consolidation, similar to lesions at 21 DPI.

Supplemental Figure 2

a

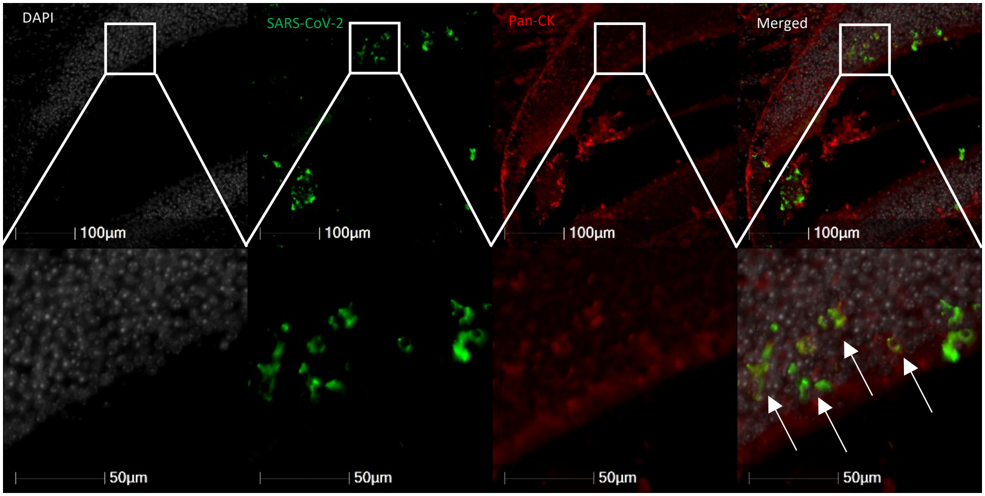

Supplemental Figure 2a. Representative images show SARS-CoV-2 infected respiratory epithelial cells as arrows pointed. (n=1)

b

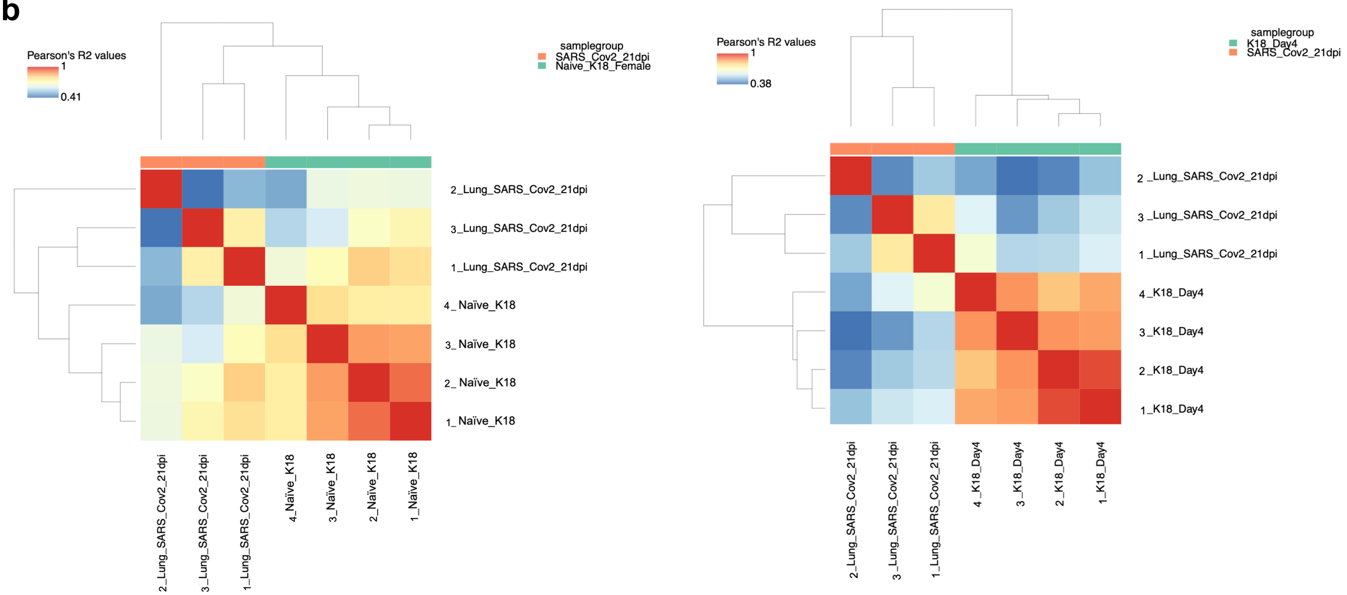

Supplemental Figure 2b. Heatmap to show sample to sample correlation. Two sample groups are presented—SARS CoV2 21 DPI (orange) and Naïve K18 and K18 4 DPI (green). Correlations were determined using all genes and a Pearson Correlation Coefficient (PCC). Color indicates PCC with 1 as the darkest red and 0.41 and 0.38 as the darkest blue. Figures represent linear relationship between mice. Representative heatmap for the CoV2 K18 21 DPI and K18 Naïve mice (left panel) and CoV2 K18 21 DPI and CoV2 K18 4 DPI mice (right panel).

Supplemental Figure 3

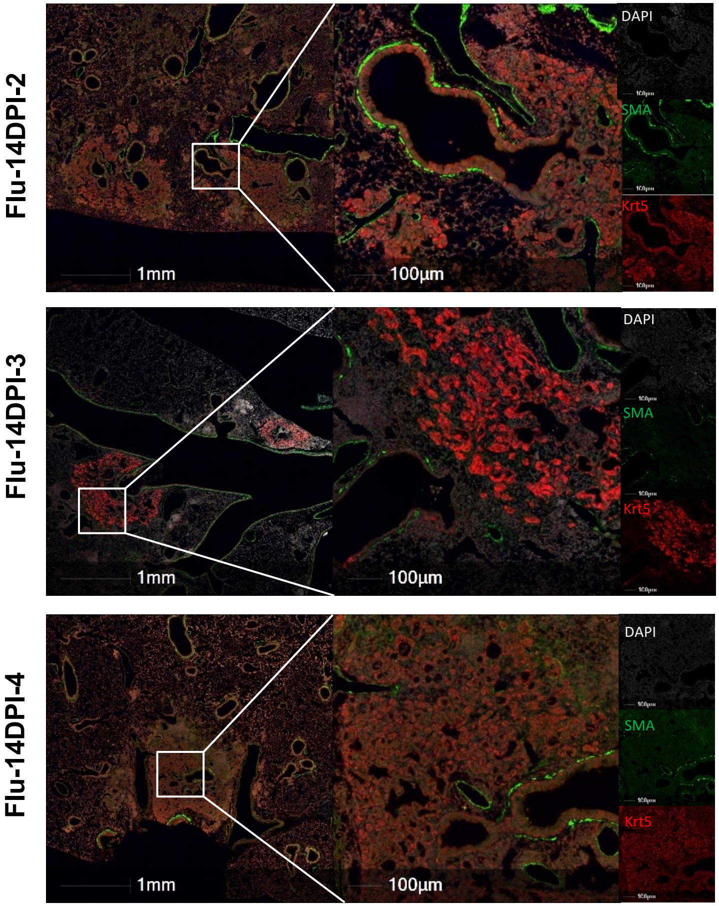

Supplemental Figure 3. Representative images show Krt5+ pod structure in lungs of each of mice infected with sublethal Flu dose at 14 DPI.

Supplemental Figure 4

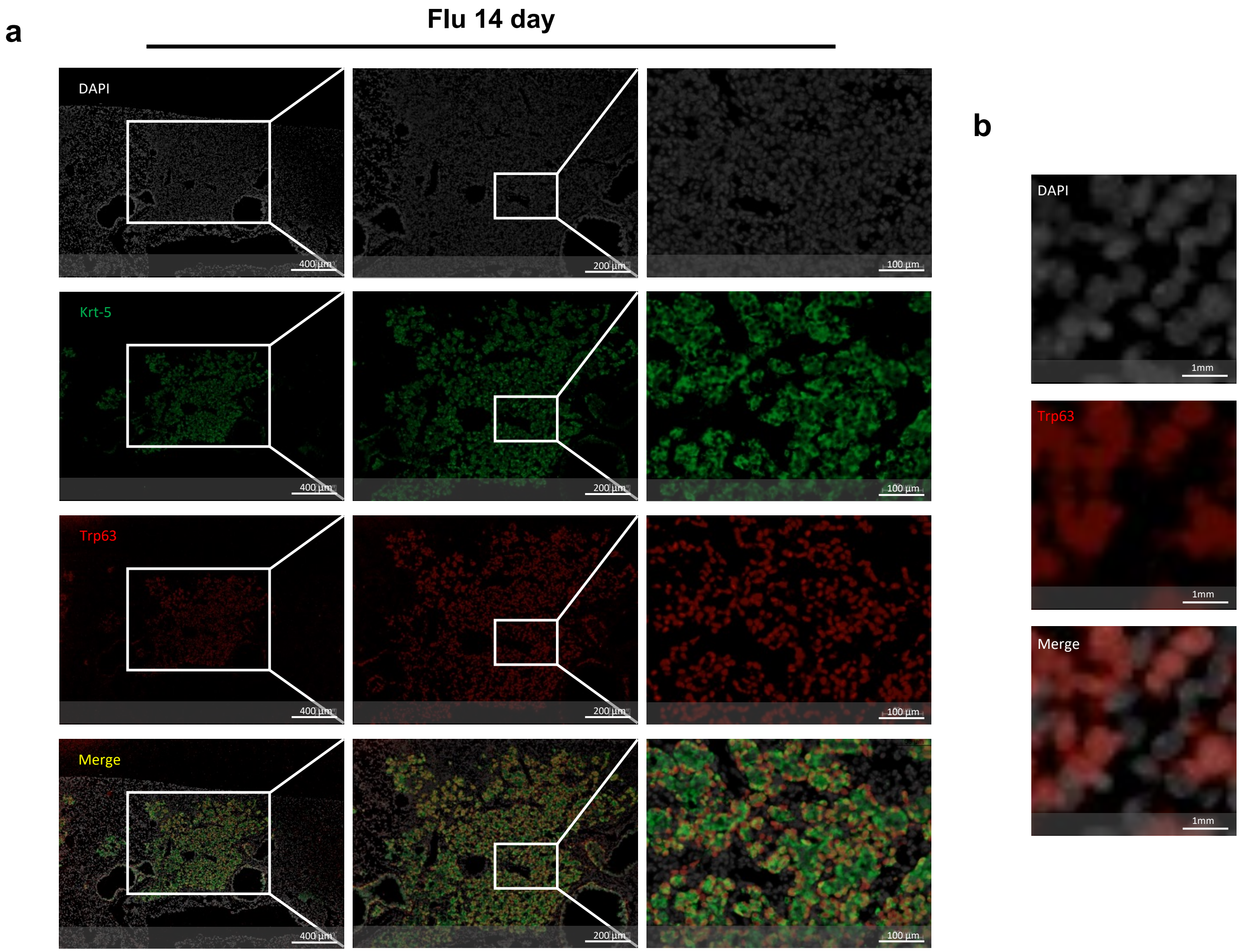

Supplemental Figure 4. Representative image shows the colocalization of Krt-5 and Trp-63 in pulmonary krt5+ "pod" structure of Flu infected mice at 14 DPI. (a) Lung sections are stained by DAPI (White), Krt-5 (green) and Trp63 (Red). At 14 DPI, Flu infected mouse exhibits Krt5+ "pods" in the lung. The Krt-5 signals colocalize with Trp63 (the highest magnification image in lowest panel). (b) The Trp63 signals colocalize with DAPI.

Supplemental Figure 5

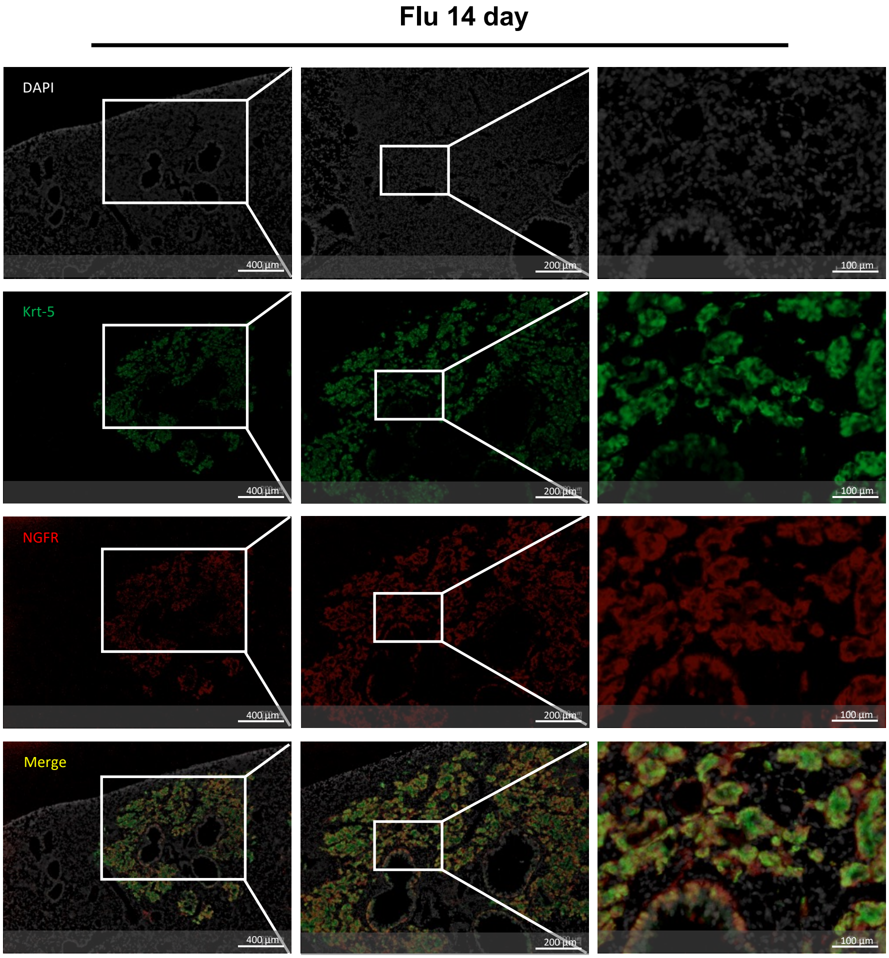

Supplemental Figure 5. Representative image shows the colocalization of Krt-5 and NGFR in pulmonary krt5+ "pod" structure of Flu infected mice at 14 DPI. Lung sections are stained by DAPI (White), Krt-5 (green) and NGFR (Red). At 14 DPI, Flu infected mouse exhibits Krt5+ "pods" in the lung. The Krt-5 signals colocalize with NGFR (the highest magnification image in lowest panel).

Supplemental Figure 6

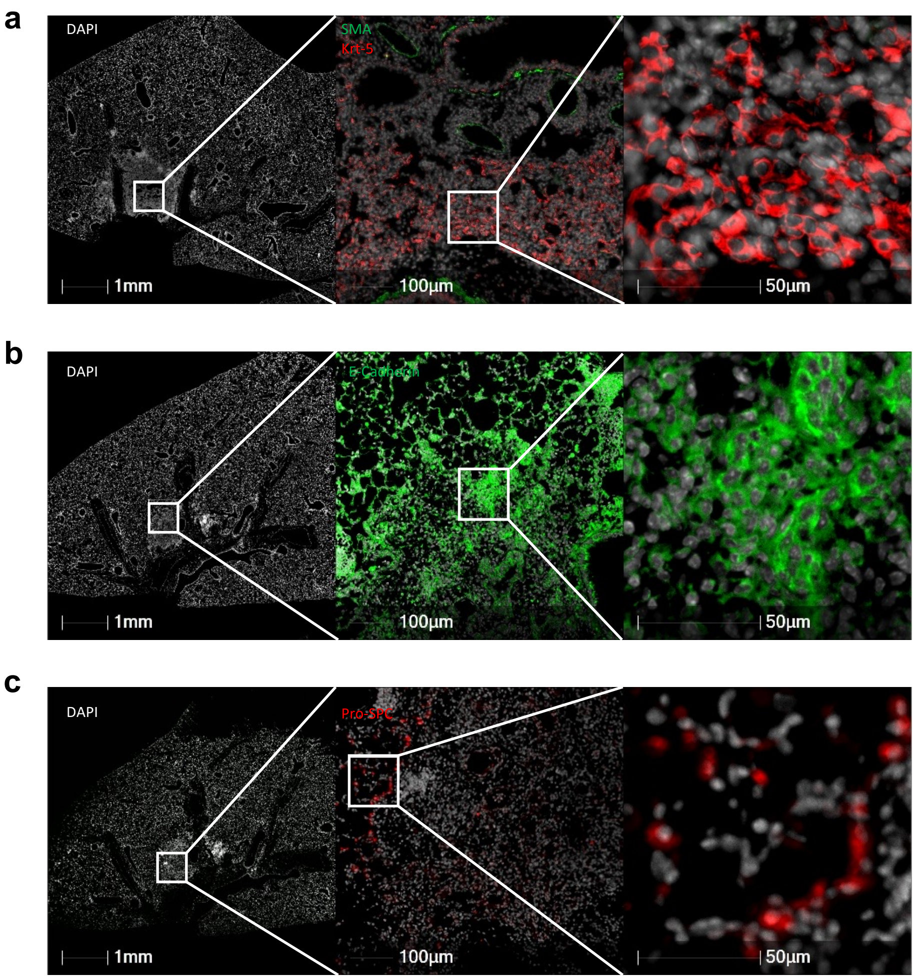

Supplemental Figure 6. Serial sections show the presence of AT1 cells in pulmonary krt-5+ "pod" area and AT2 cells at the edge of pulmonary krt5+ pod area at 14 days post Flu infection (n=4). a-c) Representative images of Krt-5, E-cad, and pro-SPC IF staining. Similar region of lung as determined by DAPI staining. (a) Krt-5+ pod is located by Krt5 fluorescence staining. (b) AT1 cells are stained by E-cad. (c) pro-SPC staining reveals AT2 cells locate at the edge of Krt-5+ pod.

Supplemental Figure 7

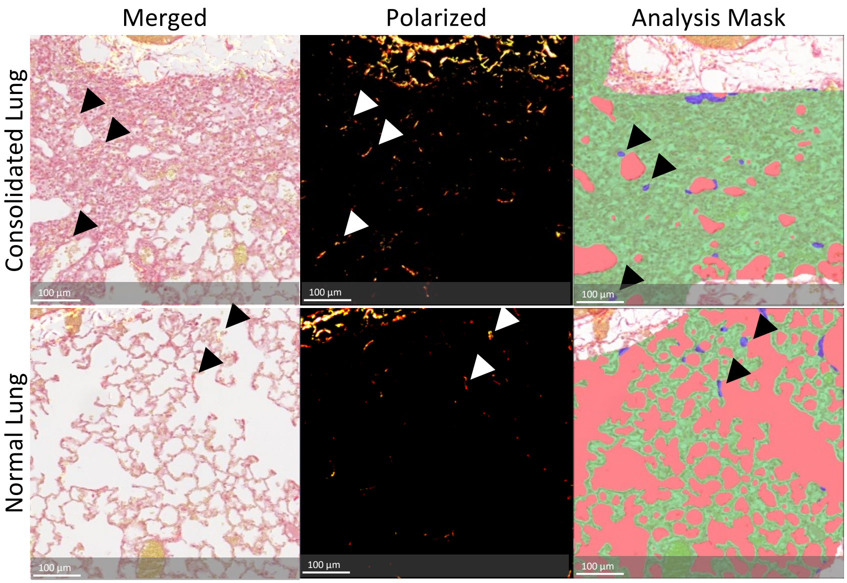

Supplemental Figure 7. Comparison of Fibrosis in Consolidated Lung versus Normal lung in Flu and COVID. Picrosirius Red staining for the comparison and quantification of fibrosis in regions of SARS-CoV-2- and Flu-associated pulmonary consolidation. **Left**) PSR staining in combination with polarized light microscopy identifies collagen by red staining and birefringence on merged images (brightfield and polarized, black arrows). **Middle**) Birefringence of collagen (white arrows) is easily seen when viewed with only polarized light and differentiates from background red staining of the merged image. **Right**) Quantification was performed with pattern recognition software trained to recognize the dual expression of red staining and birefringence (blue, black arrows). Regions of interest were drawn to exclude areas of the lung that normally contain collagen within these regions (large vessels and airways). Red = glass, green= Background.

Supplemental Figure 8

a

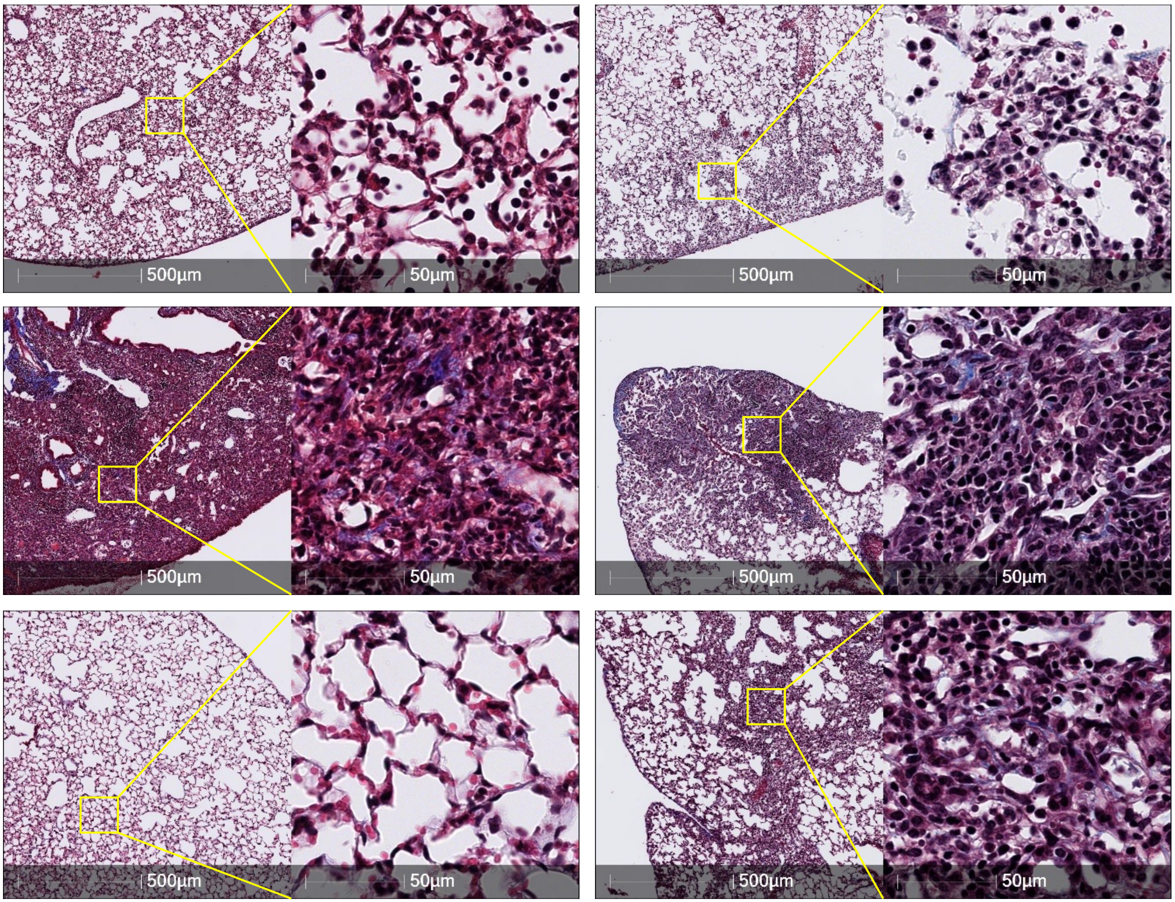

b

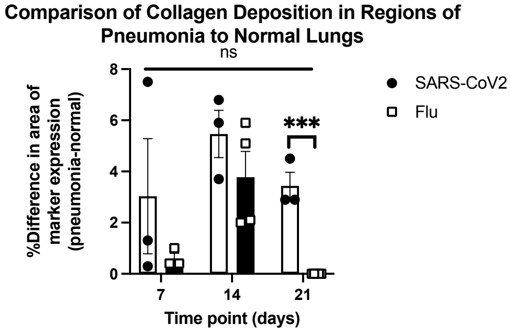

Supplemental Figure 8: Collagen deposited in Flu mouse model and COVID mouse model. (a) Masson‘s trichrome staining showing collagen deposition in lungs of 1 x10^4 $TCID_{50}$ SARS-CoV-2-infected *K18* mice at 7, 14 and 21 DPI and Flu infected mice at 5, 7, 10 and 14 DPI. (**b)** Comparison of collagen deposition in regions of consolidated lung to normal lung in the same Flu-infected and COVID mice. Data are shown as mean ± SEM. Two-way analysis of variance (ANOVA) was used to compare collagen deposition level changes over time. One-tailed unpaired Student's t-test was performed to test the difference between two groups at one time point. * * * indicates $p < 0.001$.

Supplemental Figure 9

Method of histopathologic quantification of Krt5, smooth muscle actin, and collagen deposition in regions of consolidation. Lung, influenza 14 DPI, region of consolidation. Computer software was used to quantify the deposition of collagen (a, blue), SMA (b, green), or Krt-5 (c, red) in regions of consolidation and normal lung. Detection of each was determined based on intensity of staining in brightfield (collagen) or fluorescence (SMA & Krt-5). The same region of consolidation (box, top right) is used to demonstrate the analysis results for each marker. Arrows highlight foci of marker detection in paired images. Data generated from these analysis for all normal and consolidated regions are shown in graphs below (mean ± SEM).

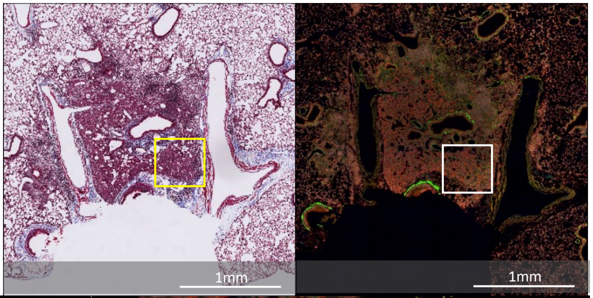

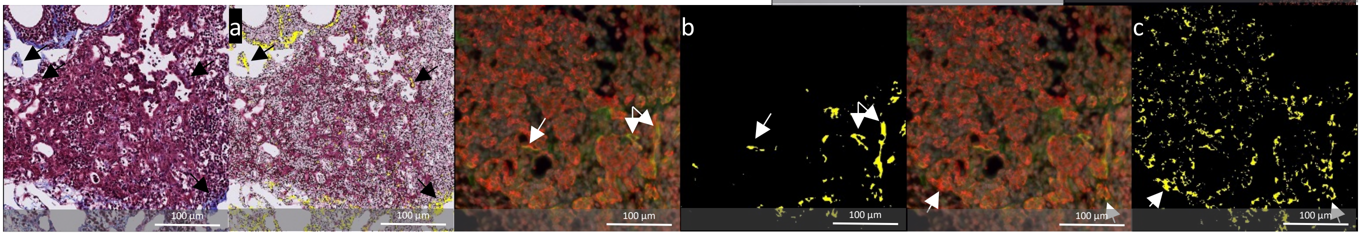

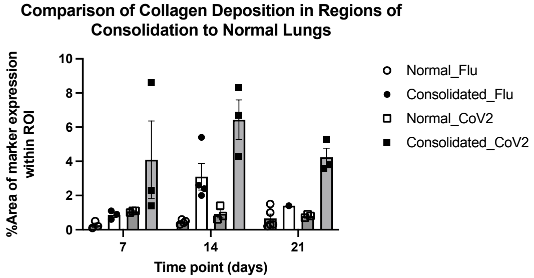

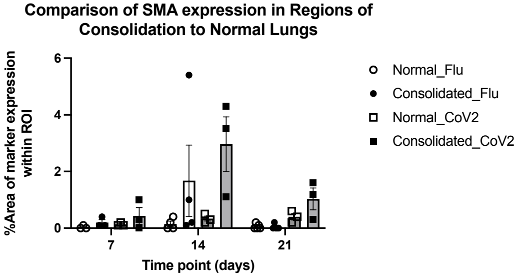

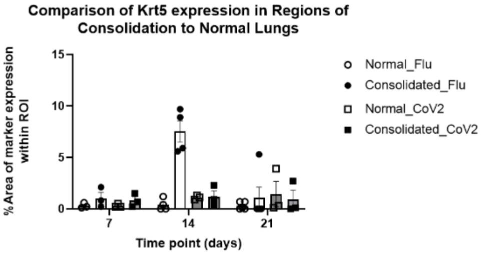

Supplemental Figure 10

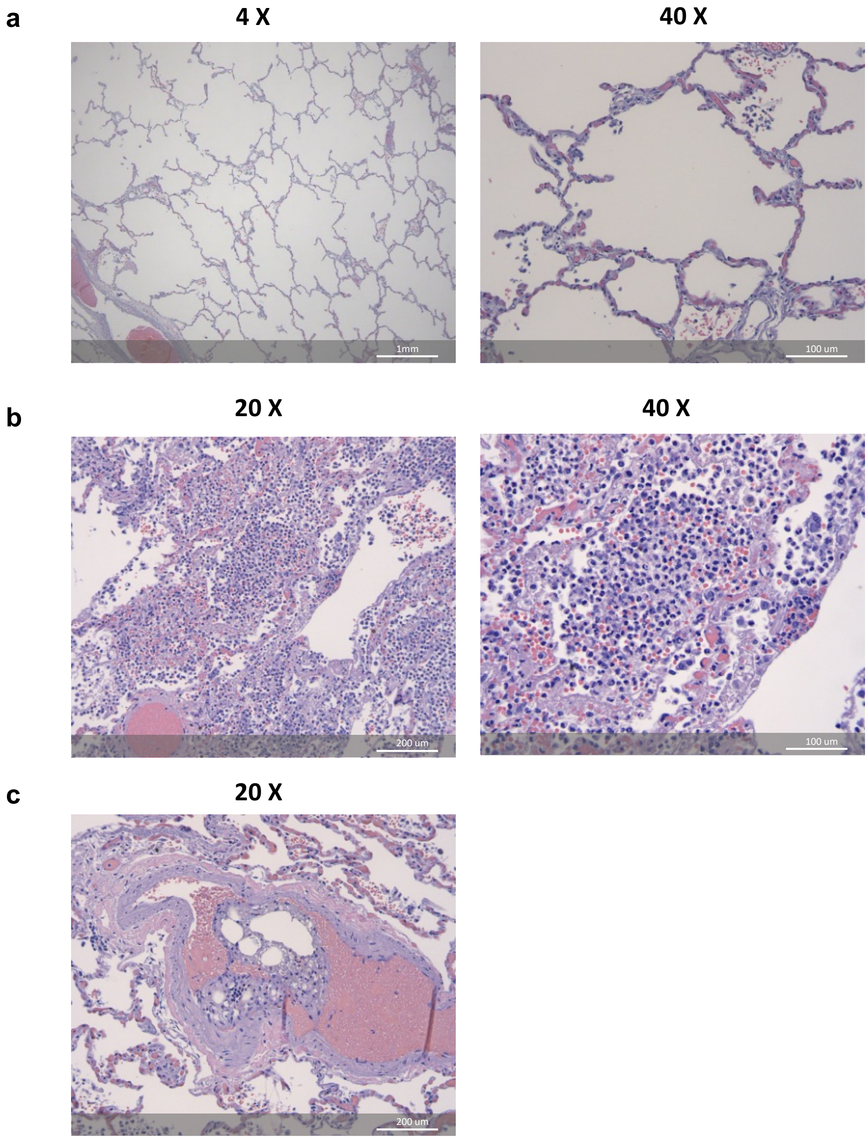

Supplemental Figure 10. Representative histopathology of COVID in human patient 3.
(a) Emphysematous changes characterized by coalescing and expanded alveolar space. (b) Organizing pneumonia with fibrocollagenous aggregates ("Masson bodies") plugging the alveoli. (c) Fat embolus in a small artery.

Supplemental Figure 11

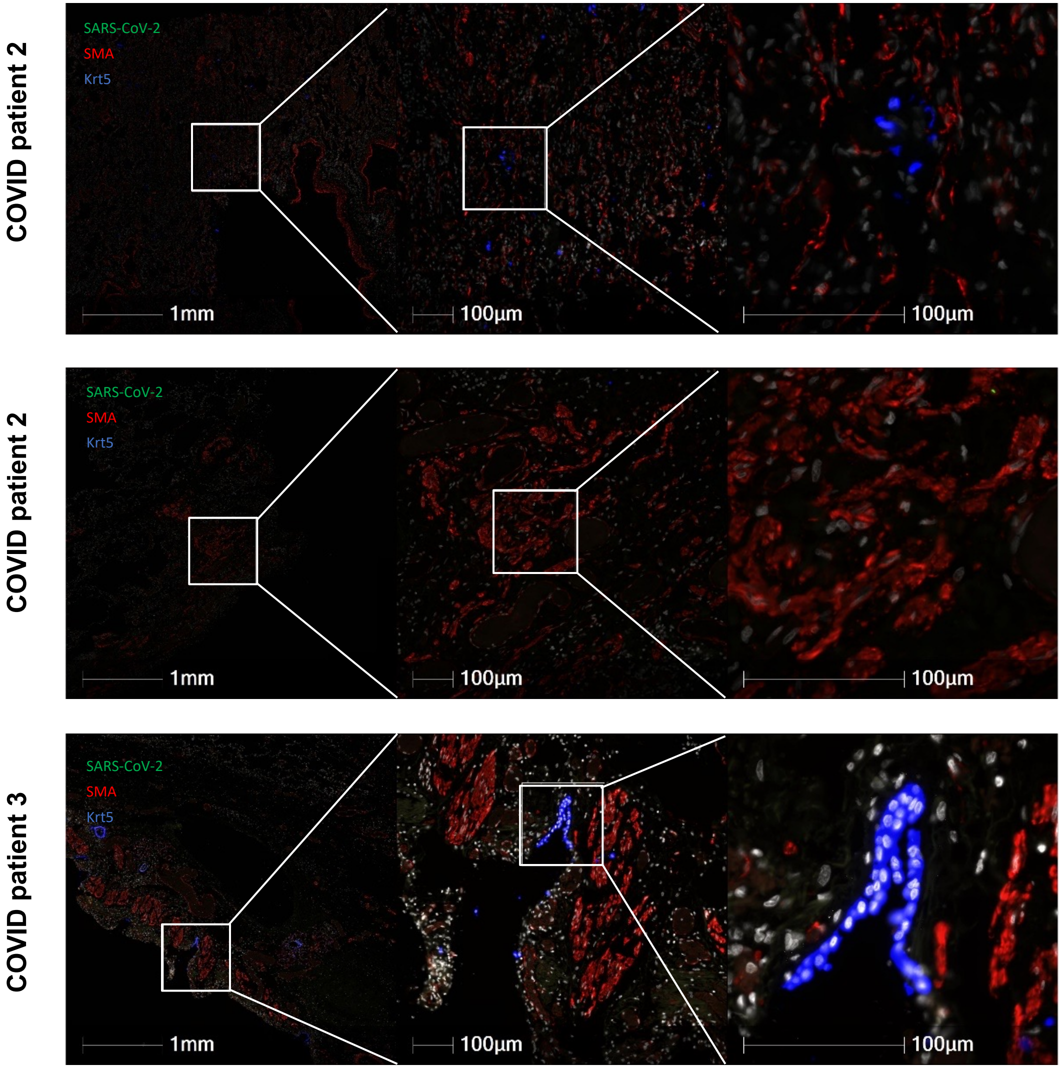

Supplemental Figure 11. No Krt5+ progenitor cell "pod" in COVID human patient 2 and patient 3. Krt5+ cells are only found in the basal layer of airways without any evidence of proliferation.

Supplemental Figure 12

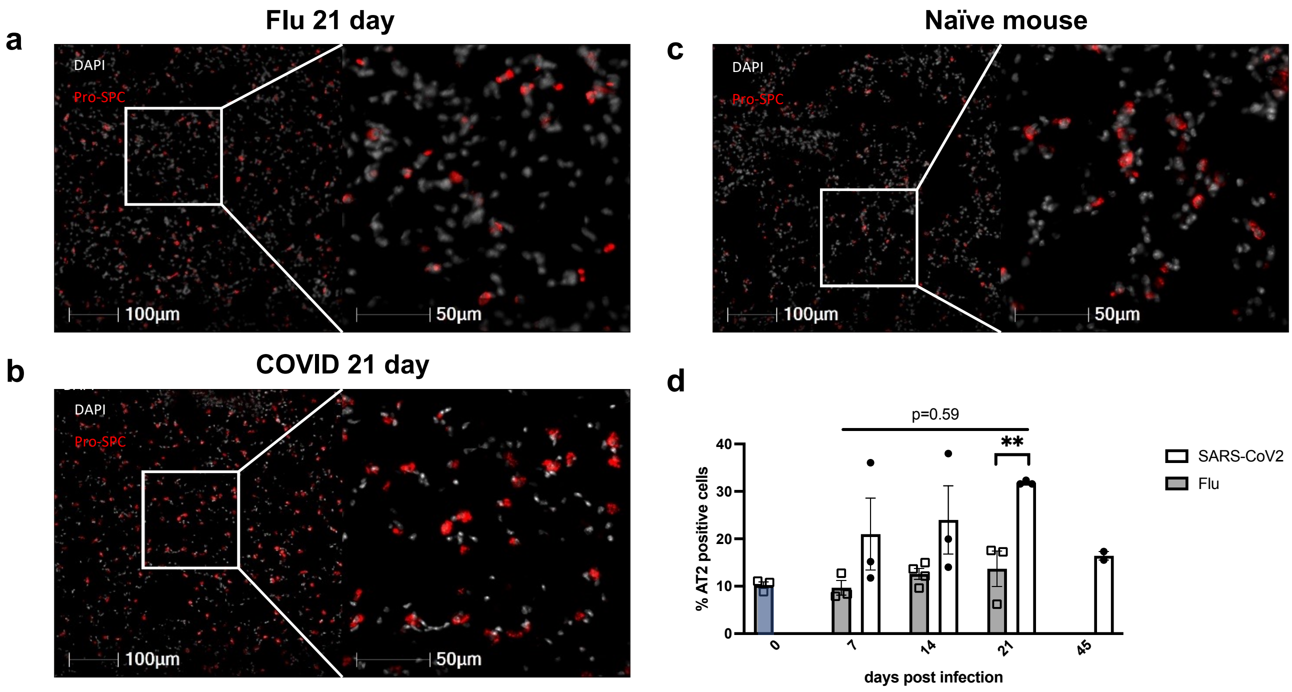

Supplemental Figure 12. Quantification of AT2 cells in the lungs of SARS-CoV-2- and Flu- infected mice. Lung sections are from naïve B6 mice (n = 3), SARS-CoV2-infected *z* mice at 7 (n = 3) , 14 (n = 3), 21 (n = 3) and 45 (n = 2)DPI, Flu-infected B6 mice at 7 (n = 3) and 14 (n = 4) DPI. a-c) Representative image of AT2 cells in the lung of mice at 21 days post flu infection, 21 days post SARS-COV2 infection and naïve mice respectively. (d) Quantified data for the percentage of AT2 cells among total pulmonary cells in the lung tissue. AT2 cells are counted by Halo software. Mixed-effect analysis is used for flu-SARS comparison. Two-tailed unpaired t test is performed to compare the difference at 21 DPI. ** indicates $p < 0.01$.

Supplementary Table 1. Mice information

Strain	Sex	Sample Size	Age/ week	Inoculum	Infection Dose	Time point /DPI	Application
K18-hACE2 +/-	Male and Female	3	28	N/A	N/A	0	Histology
K18-hACE2 +/-	Male	1	16	SARS-CoV-2(WA1/2020)	1.0X 10^4/ TCID 50	3	Histology
K18-hACE2 +/-	Male and Female	3	6-10	SARS-CoV-2(WA1/2020)	2.0X 10^5/ TCID 50	7	Body weight
K18-hACE2 +/-	Male and Female	3	12-16	SARS-CoV-2(WA1/2020)	1.0X 10^4/ TCID 50	7	Histology
K18-hACE2 +/-	Male and Female	3	12-16	SARS-CoV-2(WA1/2020)	1.0X 10^4/ TCID 50	14	Histology
K18-hACE2 +/-	Male and Female	3	10	SARS-CoV-2(WA1/2020)	1.0X 10^4/ TCID 50	21	Body weight/Histology /Viral load/Bulk RNA seq
K18-hACE2 +/-	Female	2	6-10	SARS-CoV-2(WA1/2020)	1.0X 10^4/ TCID 50	45	Histology
C57BL/6J	Male	3	6-8	H1N1 A/PR/8/34 (PR8)	50 PFU	7	Histology Staining
C57BL/6J	Male	4	6-8	H1N1 A/PR/8/34 (PR8)	50 PFU	14	Histology Staining
C57BL/6J	Male	5	6-8	H1N1 A/PR/8/34 (PR8)	50 PFU	21	Histology staining
K18-hACE2 +/-	Female	4	8	N/A	N/A	0	Bulk RNA seq
K18-hACE2 +/-	Female	3	6-10	SARS-CoV-2(WA1/2020)	2.0X 10^5/ TCID 50	4	Bulk RNA seq
K18-hACE2 +/-	Female	3	6-10	SARS-CoV-2(WA1/2020)	2.0X 10^5/ TCID 50	6	Bulk RNA seq
K18-hACE2 +/-	Female	3	8-10	H1N1 A/PR/8/34 (PR8)	50 PFU	4	Bulk RNA seq
K18-hACE2 +/-	Female	3	8-10	H1N1 A/PR/8/34 (PR8)	50 PFU	6	Bulk RNA seq
K18-hACE2 +/-	Female	1	12	SARS-CoV-2(WA1/2020)	2.0X 10^5/ TCID 50	4	Single cell RNA seq
K18-hACE2 +/-	Female	2	12	H1N1 A/PR/8/34 (PR8)	50 PFU	4	Single cell RNA seq
K18-hACE2 +/-	Female	2	12	H1N1 A/PR/8/34 (PR8)	50 PFU	6	Single cell RNA seq

DPI: Days post infection.

Supplemental Table 2. Statistically significant differentially expressed pathways between influenza and SARS-CoV-2-infected lungs at day 4 post infection.

NAME	GS<br>follow link to MSigDB	GS DETAILS	Set Size	ES	Normalized_Enrichement_Score	NOM p-val	FDR q-val	Pvalue	RANK AT MAX	LEADING EDGE
HALLMARK_INTERFERON_GAMMA_RESPONSE	HALLMARK_INTERFERON_GAMMA_RESPONSE	Details ...	157	0.656053	3.912263	0	0	0	1218	tags=75%, list=24%, signal=96%
HALLMARK_TNFA_SIGNALING_VIA_NFKB	HALLMARK_TNFA_SIGNALING_VIA_NFKB	Details ...	146	0.639828	3.768104	0	0	0	1144	tags=68%, list=23%, signal=86%
HALLMARK_INFLAMMATORY_RESPONSE	HALLMARK_INFLAMMATORY_RESPONSE	Details ...	138	0.611286	3.589829	0	0	0	1190	tags=69%, list=24%, signal=88%
HALLMARK_INTERFERON_ALPHA_RESPONSE	HALLMARK_INTERFERON_ALPHA_RESPONSE	Details ...	86	0.642152	3.470372	0	0	0	904	tags=67%, list=18%, signal=81%
HALLMARK_IL6_JAK_STAT3_SIGNALING	HALLMARK_IL6_JAK_STAT3_SIGNALING	Details ...	64	0.673658	3.296526	0	0	0	506	tags=56%, list=10%, signal=62%
HALLMARK_ALLOGRAFT_REJECTION	HALLMARK_ALLOGRAFT_REJECTION	Details ...	112	0.581183	3.239597	0	0	0	1189	tags=64%, list=24%, signal=82%
HALLMARK_P53_PATHWAY	HALLMARK_P53_PATHWAY	Details ...	95	0.492757	2.692231	0	0	0	1203	tags=54%, list=24%, signal=69%
HALLMARK_IL2_STAT5_SIGNALING	HALLMARK_IL2_STAT5_SIGNALING	Details ...	107	0.421067	2.389516	0	0	0	918	tags=41%, list=18%, signal=49%
HALLMARK_COMPLEMENT	HALLMARK_COMPLEMENT	Details ...	104	0.410134	2.291471	0	1.43E-04	0.001	1217	tags=50%, list=24%, signal=65%
HALLMARK_KRAS_SIGNALING_UP	HALLMARK_KRAS_SIGNALING_UP	Details ...	98	0.408836	2.256511	0	1.29E-04	0.001	891	tags=38%, list=18%, signal=45%
HALLMARK_APOPTOSIS	HALLMARK_APOPTOSIS	Details ...	80	0.41056	2.158816	0	4.88E-04	0.004	1387	tags=52%, list=27%, signal=71%
HALLMARK_MYC_TARGETS_V2	HALLMARK_MYC_TARGETS_V2	Details ...	30	0.499773	2.103931	0	5.54E-04	0.005	1896	tags=87%, list=38%, signal=138%
HALLMARK_COAGULATION	HALLMARK_COAGULATION	Details ...	60	0.425369	2.067774	0	6.93E-04	0.007	1395	tags=53%, list=28%, signal=73%

Supplemental Table 3. Statistically significant differentially expressed pathways between influenza and SARS-CoV-2-infected lungs at day 6 post infection

NAME	GS<br>follow link to MSigDB	GS DETAILS	SIZE	ES	Normilized_ Enrichment_ Score	NOM p-val	FDR q-val	FWER p-val	RANK AT MAX	LEADING EDGE
HALLMARK_TNFA_SIGNALING_VIA_NFKB	HALLMARK_TNFA_SIGNALING_VIA_NFKB	Details ...	106	0.606244	3.1957	0	0	0	1332	tags=83%, list=30%, signal=116%
HALLMARK_P53_PATHWAY	HALLMARK_P53_PATHWAY	Details ...	78	0.512818	2.609483	0	0	0	1252	tags=65%, list=28%, signal=89%
HALLMARK_KRAS_SIGNALING_UP	HALLMARK_KRAS_SIGNALING_UP	Details ...	89	0.497607	2.554852	0	0	0	829	tags=54%, list=19%, signal=65%
HALLMARK_EPITHELIAL_MESENCHYMAL_TRANSITION	HALLMARK_EPITHELIAL_MESENCHYMAL_TRANSITION	Details ...	77	0.472535	2.381339	0	0	0	1375	tags=66%, list=31%, signal=94%
HALLMARK_INFLAMMATORY_RESPONSE	HALLMARK_INFLAMMATORY_RESPONSE	Details ...	100	0.43981	2.325041	0	0	0	756	tags=49%, list=17%, signal=58%
HALLMARK_IL6_JAK_STAT3_SIGNALING	HALLMARK_IL6_JAK_STAT3_SIGNALING	Details ...	42	0.485532	2.065453	0	0	0	1159	tags=57%, list=26%, signal=77%
HALLMARK_TGF_BETA_SIGNALING	HALLMARK_TGF_BETA_SIGNALING	Details ...	24	0.486825	1.854932	0.003378	0.009194	0.021	1539	tags=79%, list=35%, signal=120%
HALLMARK_APOPTOSIS	HALLMARK_APOPTOSIS	Details ...	67	0.316087	1.584815	0.014423	0.073386	0.172	826	tags=42%, list=19%, signal=51%
HALLMARK_APICAL_JUNCTION	HALLMARK_APICAL_JUNCTION	Details ...	69	0.308623	1.532568	0.009346	0.092613	0.231	776	tags=32%, list=17%, signal=38%
HALLMARK_UV_RESPONSE_UP	HALLMARK_UV_RESPONSE_UP	Details ...	47	0.334876	1.503433	0.035897	0.105078	0.276	1135	tags=49%, list=25%, signal=65%
HALLMARK_COMPLEMENT	HALLMARK_COMPLEMENT	Details ...	77	0.275105	1.387579	0.022727	0.193682	0.487	440	tags=19%, list=10%, signal=21%

Supplementary Table 4. Animal Information for Nonhuman Primate Comparators

Species	Sex	Age	Weight	Inoculum	Timepoint
Chlorocebus aethiops	Male	7.35	4.2	RSV	2 WPI
Chlorocebus aethiops	Male	17.34	5.45	SHAM	4 WPI
Chlorocebus aethiops	Male	16.33	7.45	SARS-CoV-2	4 WPI
Chlorocebus aethiops	Male	16.3	6.9	SARS-CoV-2	4 WPI
Chlorocebus aethiops	Female	16.29	3.85	SARS-CoV-2	4 WPI
Chlorocebus aethiops	Female	16.28	4.25	SARS-CoV-2	2 WPI

WPI: Weeks post SARS-CoV-2 infection.

Supplementary Table 5. Human Case Information

Patient No.	1	2	3
**Patient ID**	UMAU20-00028	TMC-20-1(A14-20-1)	TMC-21-3
**Age**	78 years	58 years	86 years
**Gender**	female	male	male
**Comorbidities**	end-stage renal disease, type 2 diabetes and obesity	hypertension, type 2 diabetes mellitus, hepatitis C virus and chronic kidney disease	bilateral sacral fracture, atrial fibrillation, heart failure, hypertension, hyperlipidemia, stage 3 chronic kidney disease, cerebrovascular disease, peripheral artery disease, esophageal dilation, Parkinson disease, benign prostatic hyperplasia, and smoking
**duration after test postive**	2 day	32 days	17 days
**histology changes**	Her lungs exhibited early changes of COVID-19 disease and thrombosis. These included focal hemorrhage and severe alveolar edema with early hyaline membrane formation. She had numerous clots in the pulmonary vessels and severely elevated d-dimer levels (10,020 ng/ml; normal <250 ng/ml). The edema and hyaline membranes are early changes associated with diffuse alveolar damage. Case report can be found in [1].	extensive acute bronchopneumonia with focal bacteria colonies of cocci, multifocal organizing pneumonia with predominantly fibrosing and focally fibrinous features, which were consistent with a background COVID-19 infection.Additional significant findings included hypertrophic cardiomegaly, severe calcific atherosclerosis of the coronary arteries, centrilobular and midzonal necrosis of the liver consist with severe ischemic necrosis, glomerulosclerosis and arteriosclerosis of the kidneys, and diffuse mediastinal, hilar and mesenteric reactive lymphadenopathy. Case report can be found in [1].	aspiration pneumonia, emphysema, bone marrow, fat emboli, hypertensive hypertrophic cardiomyopathy, acute peritonitis, necrosis of the distal esophagus, glomerulosclerosis, and acute tubular necrosis. (Figures are attached as Supplemental Figure 10)

**complications**	cardiac arrest	end-stage renal failure requiring dialysis, and anoxic brain injury secondary to hypoxia	a right thigh hematoma and drop in hemoglobin, coffee ground emesis, pneumatosis of the stomach, pneumoperitoneum,necrosis of the distal esophagus, continued intraabdominal abscess, and bleeding from the spleen
**progenitor cell staining description**	Scatttered krt-5 positive cells are found in SARS-CoV2 deposit area. No "pod" structure.	Scatttered krt-5 positive cells are found in fibrosis area. No "pod" structure.	Very few krt-5 positive cells are found in fibrosis area. No "pod" structure.

Reference:

1. Liu, F., et al., *SARS-CoV-2 Infects Endothelial Cells In Vivo and In Vitro.* Front Cell Infect Microbiol, 2021. **11**: p. 701278.

Supplementary Table 6. Antibody Information

Anti-SARS	Anti-SARS-CoV	1: 1000	BEI Resources	# NR-10361
Anti-CD206	Anti-mouse	1: 50	R & D	# AF2535
Anti-SMA	Anti-mouse	1: 100	Abcam	# ab5694-100
Anti-Krt-5	Anti-mouse	1: 1000	BioLegend	# 905901
Anti-SMA	Anti-human	1: 100	Agilent	# M085129-2
Anti-Krt-5	Anti-human	1: 50	Abcam	# ab17130
Anti-pro SPC	Anti-mouse	1: 500	Seven Hills Bioreagents	# WRAB-9337
Anti-trp63	Anti-mouse	1: 200	Cell Signaling Technology	# 13109
Anti-NGFR	Anti-mouse	1: 100	Abcam	# ab52987
