# Supplementary material for: COVID-19 and influenza infections mediate distinct pulmonary cellular and transcriptomic changes
Source: Commun Biol. 2023 Dec 13;6:1265. doi: 10.1038/s42003-023-05626-z (PMC10719262; doi:10.1038/s42003-023-05626-z)
Supplement: Supplementary file 3 — Description of additional supplementary files [file 42003_2023_5626_MOESM3_ESM.pdf]

Description of Additional Supplementary Files

File name: Supplemental Data 1
Description: Differential gene expression of K18_COVID_21dpi_vs_K18_Naive

File name: Supplemental Data 2
Description: Differential Collagen gene expression of K18_COVID_21dpi_vs_K18_Naive

File name: Supplemental Data 3
Description: Differential gene expression of COVID_ 21dpi_vs_COVID_21dpi

File name: Supplemental Data 4
Description: Three groups comparison_Differential gene expression_COVID_4dpi

File name: Supplemental Data 5
Description: Three groups comparison_Differential gene expression_COVID_6dpi

File name: Supplemental Data 6
Description: Differential gene expression of Flu_4dpi_vs_Covid_4dpi

File name: Supplemental Data 7
Description: Differential gene expression of Flu_6dpi_vs_Covid_6dpi

File name: Supplemental Data 8
Description: Differential gene expression_scRNA_Flu K18 4 DPI vs COVID K18 4 DPI in epithelial cell

File name: Supplemental Data 9
Description: DEG analysis scRNA individual data (Flu 4 &6 DPI and COVID 4 DPI)

File name: Supplemental Data 10
Description: The source data behind the graphs in the paper
