# Supplementary material for: COVID-19 and influenza infections mediate distinct pulmonary cellular and transcriptomic changes
Source: Commun Biol. 2023 Dec 13;6:1265. doi: 10.1038/s42003-023-05626-z (PMC10719262; doi:10.1038/s42003-023-05626-z)
Supplement: Supplementary file 7 — Reporting Summary [file 42003_2023_5626_MOESM7_ESM.pdf]

nature portfolio

Corresponding author(s): Xuebin Qin

Last updated by author(s): Oct 2, 2023

Reporting Summary

Nature Portfolio wishes to improve the reproducibility of the work that we publish. This form provides structure for consistency and transparency in reporting. For further information on Nature Portfolio policies, see our Editorial Policies and the Editorial Policy Checklist.

Statistics

For all statistical analyses, confirm that the following items are present in the figure legend, table legend, main text, or Methods section.

n/a	Confirmed	
☐	☒	The exact sample size (*n*) for each experimental group/condition, given as a discrete number and unit of measurement
☐	☒	A statement on whether measurements were taken from distinct samples or whether the same sample was measured repeatedly
☐	☒	The statistical test(s) used AND whether they are one- or two-sided<br>*Only common tests should be described solely by name; describe more complex techniques in the Methods section.*
☐	☒	A description of all covariates tested
☐	☒	A description of any assumptions or corrections, such as tests of normality and adjustment for multiple comparisons
☐	☒	A full description of the statistical parameters including central tendency (e.g. means) or other basic estimates (e.g. regression coefficient) AND variation (e.g. standard deviation) or associated estimates of uncertainty (e.g. confidence intervals)
☐	☒	For null hypothesis testing, the test statistic (e.g. *F*, *t*, *r*) with confidence intervals, effect sizes, degrees of freedom and *P* value noted<br>*Give P values as exact values whenever suitable.*
☒	☐	For Bayesian analysis, information on the choice of priors and Markov chain Monte Carlo settings
☒	☐	For hierarchical and complex designs, identification of the appropriate level for tests and full reporting of outcomes
☒	☐	Estimates of effect sizes (e.g. Cohen's *d*, Pearson's *r*), indicating how they were calculated

Our web collection on statistics for biologists contains articles on many of the points above.

Software and code

Policy information about availability of computer code

Data collection	N/A
Data analysis	Bulk RNA seq and single cell RNA seq is analyzed by R. Body weights, viral load, pathology is analyzed by Graphpad Prism. Fluorescence and inflammation level is analyzed by HALO.

For manuscripts utilizing custom algorithms or software that are central to the research but not yet described in published literature, software must be made available to editors and reviewers. We strongly encourage code deposition in a community repository (e.g. GitHub). See the Nature Portfolio guidelines for submitting code & software for further information.

Data

Policy information about availability of data

All manuscripts must include a data availability statement. This statement should provide the following information, where applicable:

- Accession codes, unique identifiers, or web links for publicly available datasets
- A description of any restrictions on data availability
- For clinical datasets or third party data, please ensure that the statement adheres to our policy

Provide your data availability statement here.

Research involving human participants, their data, or biological material

Policy information about studies with human participants or human data. See also policy information about sex, gender (identity/presentation), and sexual orientation and race, ethnicity and racism.

Reporting on sex and gender	One female patient and two male patients were included as human data. We designed to included both sex in study design. Sex/ gender was determined by self-reporting or assigned. Human case summary is reported as supplemental table 12.
Reporting on race, ethnicity, or other socially relevant groupings	The socially constructed or socially relevant categorization variables are not used in the manuscript.
Population characteristics	The detailed information is summarized in supplemental table 12.
Recruitment	Participants are recruited by the disease severity. Severe COVID patients showing pulmonary disease are further included in the study.
Ethics oversight	Tulane University approved the study protocol.

Note that full information on the approval of the study protocol must also be provided in the manuscript.

Field-specific reporting

Please select the one below that is the best fit for your research. If you are not sure, read the appropriate sections before making your selection.

☒ Life sciences ☐ Behavioural & social sciences ☐ Ecological, evolutionary & environmental sciences

For a reference copy of the document with all sections, see nature.com/documents/nr-reporting-summary-flat.pdf

Life sciences study design

All studies must disclose on these points even when the disclosure is negative.

Sample size	In our previous published paper (doi: 10.7150/thno.61810. eCollection 2021.). We performed a comprehensive test on both male and female K18 mice infected with SARS-CoV-2. Based on these data, we didn't do statistical methods to predetermine sample size.
Data exclusions	No data were excluded from the analysis.
Replication	Since our focus is long-term covid, we collected the tissue as long as 21 days and 45 days post infection. Together with the limitation of BSL3 facility, it is not practical to replicate the experiment for several times. To increase the vadility and reproductivity, we tested the samples from multiple time points such as 7, 14, 21, and 45 days post infection.
Randomization	Randomization is not relevant to our study. To increase the validity, the virus is diluted from the same stock.
Blinding	The pathologist performed the histology analysis is blinded to the animal grouping.

Reporting for specific materials, systems and methods

We require information from authors about some types of materials, experimental systems and methods used in many studies. Here, indicate whether each material, system or method listed is relevant to your study. If you are not sure if a list item applies to your research, read the appropriate section before selecting a response.

Materials & experimental systems

n/a	Involved in the study	
☐	☒	Antibodies
☒	☐	Eukaryotic cell lines
☒	☐	Palaeontology and archaeology
☐	☒	Animals and other organisms
☐	☒	Clinical data
☒	☐	Dual use research of concern
☒	☐	Plants

Methods

n/a	Involved in the study	
☒	☐	ChIP-seq
☒	☐	Flow cytometry
☒	☐	MRI-based neuroimaging

Antibodies

Antibodies used	Anti-SARS Anti-SARS-CoV 1: 1000 BEI Resources # NR-10361<br>Anti-CD206 Anti-mouse 1: 50 R & D # AF2535<br>Anti-SMA Anti-mouse 1: 100 Abcam # ab5694-100<br>Anti-Krt-5 Anti-mouse 1: 1000 BioLegend # 905901<br>Anti-SMA Anti-human 1: 100 Agilent # M085129-2<br>Anti-Krt-5 Anti-human 1: 50 Abcam # ab17130<br>Anti-pro SPC Anti-mouse 1: 500 Seven Hills Bioreagents # WRAB-9337<br>Anti-trp63 Anti-mouse 1: 200 Cell Signaling Technology # 13109<br>Anti-NGFR Anti-mouse 1: 100 Abcam # ab52987
Validation	The validation of each primary antibody is performed by manufacturers.<br>https://www.beiresources.org/Catalog/BEIPolyclonalAntiserum/NR-10361.aspx<br>https://www.rndsystems.com/products/mouse-mmr-cd206-antibody_af2535<br>https://www.abcam.com/products/primary-antibodies/alpha-smooth-muscle-actin-antibody-ab5694.html<br>https://www.biolegend.com/fr-fr/explore-new-products/keratin-5-polyclonal-chicken-antibody-purified-10957<br>https://www.agilent.com/en/product/immunohistochemistry/antibodies-controls/primary-antibodies/actin-%28smooth-muscle%29-%28concentrate%29-76542<br>https://www.abcam.com/products/primary-antibodies/cytokeratin-5-antibody-xm26-ab17130.html<br>https://www.sevenhillsbioreagents.com/products/anti-pro-sp-c-rabbit-n-terminal<br>https://www.cellsignal.com/products/primary-antibodies/p63-a-d2k8x-xp-rabbit-mab/13109<br>https://www.abcam.com/products/primary-antibodies/p75-ngf-receptor-antibody-ep1039y-bsa-and-azide-free-ab256584.html

Animals and other research organisms

Policy information about studies involving animals; ARRIVE guidelines recommended for reporting animal research, and Sex and Gender in Research

Laboratory animals	Strain Sex Sample Size Age/ week Inoculum Infection Dose Timepoint/DPI<br>K18-hACE2 +/- Male and Female 3 28 N/A N/A 0<br>K18-hACE2 +/- Male 1 16 SARS-CoV-2(WA1/2020) 1.0X 10^4/ TCID 50 3<br>K18-hACE2 +/- Male and Female 3 6-10 SARS-CoV-2(WA1/2020) 2.0X 10^5/ TCID 50 7<br>K18-hACE2 +/- Male and Female 3 12-16 SARS-CoV-2(WA1/2020) 1.0X 10^4/ TCID 50 7<br>K18-hACE2 +/- Male and Female 3 12-16 SARS-CoV-2(WA1/2020) 1.0X 10^4/ TCID 50 14<br>K18-hACE2 +/- Male and Female 3 10 SARS-CoV-2(WA1/2020) 1.0X 10^4/ TCID 50 21<br>K18-hACE2 +/- Female 2 6-10 SARS-CoV-2(WA1/2020) 1.0X 10^4/ TCID 50 45<br>C57BL/6J Male 3 6-8 H1N1 A/PR/8/34 (PR8) 50 PFU 7<br>C57BL/6J Male 4 6-8 H1N1 A/PR/8/34 (PR8) 50 PFU 14<br>C57BL/6J Male 5 6-8 H1N1 A/PR/8/34 (PR8) 50 PFU 21<br>K18-hACE2 +/- Female 4 8 N/A N/A 0<br>K18-hACE2 +/- Female 3 6-10 SARS-CoV-2(WA1/2020) 2.0X 10^5/ TCID 50 4<br>K18-hACE2 +/- Female 3 6-10 SARS-CoV-2(WA1/2020) 2.0X 10^5/ TCID 50 6<br>K18-hACE2 +/- Female 3 8-10 H1N1 A/PR/8/34 (PR8) 50 PFU 4<br>K18-hACE2 +/- Female 3 8-10 H1N1 A/PR/8/34 (PR8) 50 PFU 6<br>K18-hACE2 +/- Female 1 12 SARS-CoV-2(WA1/2020) 2.0X 10^5/ TCID 50 4<br>K18-hACE2 +/- Female 1 12 H1N1 A/PR/8/34 (PR8) 50 PFU 4
Wild animals	No wild animals are included in the study
Reporting on sex	The finding applies to both sex. No sex-based analysis is performed.
Field-collected samples	No field-collected samples are included in the study.
Ethics oversight	Tulane University

Note that full information on the approval of the study protocol must also be provided in the manuscript.

Clinical data

Policy information about clinical studies

All manuscripts should comply with the ICMJE guidelines for publication of clinical research and a completed CONSORT checklist must be included with all submissions.

Clinical trial registration	N/A
Study protocol	N/A

Data collection	N/A
Outcomes	N/A
